# How Income and Discrimination Shape the Acceptance of Newcomers in Europe: A Comparative Analysis of Native-Born and Immigrant Populations

**DOI:** 10.3390/bs14090743

**Published:** 2024-08-25

**Authors:** Nonna Kushnirovich

**Affiliations:** Ruppin Academic Center, Department of Economics and Management, Institute for Immigration and Social Integration, Emek Hefer 4025000, Israel; nonna@ruppin.ac.il

**Keywords:** immigrants, discrimination, threats, attitudes

## Abstract

The purpose of this study was to investigate how income and belonging to a discriminated group are associated with perceptions of threats posed by immigrants, and with the willingness to accept newcomers of a different/same race or ethnicity as most people of the receiving country, or newcomers who came from poor countries outside Europe. The study transcended Borjas’s theory of ‘competing and complementary’ to newcomer groups of native workers, expanding it from the economic and labor spheres to the symbolic cultural and social spheres, and extending this theory to the foreign-born European population. The study used data from the European Social Survey Round 10 Data. Three local population groups in the EU were examined: the native-born population, immigrants from non-EU countries living in the EU, and migrants from EU countries living in other EU countries. The study revealed that for native-born people, the salient factor predicting their perceived threats and willingness to accept newcomers was income, and for non-EU veteran immigrants, the salient factor was the feeling of belonging to a discriminated group. Economically disadvantaged native-born people in the EU were a group competing with newcomers. However, disadvantaged and discriminated non-EU immigrants were complementary to newcomers. The study showed that a disadvantaged group may be either competing or complementary to newcomers, depending on the origin of the group’s members rather than on the origin of newcomers.

## 1. Introduction

Europe has been experiencing waves of immigration over recent years. Almost 3.4 million permits to reside were granted in the EU in 2022 as opposed to 2.9 million in 2021, and 3.0 million permits in 2019. In 2023, 8.5% of all EU inhabitants were born outside the EU [1]. 

Willingness to receive and accept immigrants has become a hot topic of discussion in recent years, as the threat of effects on the economy and culture has fueled a reaction against globalization [2,3,4,5]. The idea that threat is the basic driver of intergroup prejudice and willingness to welcome new immigrants has been widely discussed in the literature [5,6,7,8,9,10,11,12]. However, these studies focused mostly on the threat perceptions of certain population groups (only native-born people or only immigrants), and did not examine whether migrants’ feelings of threat and their attitudes toward newcomers were shaped by the same factors and in the same manner as those of the non-migrant population. For example, the study by Pereira et al. [11] on opposition to immigration in Europe omitted immigrants and non-natives from the analysis. A few years later, Gorodzeisky and Semyonov [13] analyzed the opposition in Europe to various groups of immigrants (Muslim, Jewish, and Roma), but they also did not consider the views of the immigrants themselves and their perception of threats from newcomers. The study by Jeannet [14], focusing on attitudes toward immigrants from other EU countries (those who moved from East to West Europe), also restricted the sample to individuals without an immigrant background.

Other studies investigated perceptions only of immigrant groups in Europe. For example, Van der Zwan, Bles and Lubbers [15], who focused on feelings of threat amongst immigrants in Europe from newcomers, included only first-generation and second-generation migrants and did not include people with no immigrant background. None of these studies compared perceptions of native-born Europeans with perceptions of immigrants from other EU countries and non-EU immigrants. Such a comparison is important, since previous studies showed that the native population demonstrated fewer sentiments about newcomers than persons with a migrant background [16,17]. Outside Europe, only rare studies explored the attitudes and behavioral intentions of immigrants toward other immigrant groups [18].

Various immigrant groups may be perceived differently by the local population [19]. Recent research revealed that attitudes towards immigrants in Europe vary by ethnic or religious origin, with more negative attitudes towards immigrants from Muslim countries [7,13,20]. In Britain, attitudes towards White and culturally similar immigrants from Australia and Western Europe were better than attitudes toward non-White and culturally dissimilar immigrants from India and South Asia [21]. Immigrants from the same racial or ethnic group as the country’s majority were preferred over those from other ethnic groups or poorer European countries, who, in turn, were preferred over those from poorer countries outside of Europe [22]. In the USA, fewer favorable perceptions of immigrants from Iraq, Somalia, and Sudan were found [23]; in Canada, Sudanese immigrants were more likely to face rejection [24].

A range of studies explored how restrictive immigrant policies and prejudice shaped the feeling of discrimination of immigrants when discrimination was an explained variable [25,26,27]. However, the opposite effect of feeling discrimination on acceptance of or opposition to newcomers remained under-investigated. Additional determinants of anti- or pro-immigrant sentiments are income, employment, or other terms of the personal economic situation [2,13,25,26,28,29]. Since income provides people with a sense of stability, it may decrease feelings of threat and sometimes even buffer the negative effect of feelings of discrimination [30]. However, existing studies do not investigate which determinant, income or feeling of being discriminated against, is more important in shaping acceptance/opposition to newcomers of different population groups.

This study seeks to fill these gaps. It investigates how income and belonging to a discriminated group are associated with perceptions of threat posed by immigrants, and with the willingness to accept newcomers of a different/similar race or ethnic group from most people of the receiving country, or newcomers who came from the poor countries outside Europe. The willingness of three local population groups in the EU to accept newcomers was examined: the willingness of the native-born population, of immigrants from non-EU countries living in the EU, and migrants from EU countries living in other EU countries. The study investigated whether immigrants themselves felt threatened by other immigrants, whether they were likely to allow new waves of immigrants to enter the EU, and whether their own origin and the origin of newcomers mattered. The study transcended Borjas’s [31] theory of ‘competing and complementary’ newcomer groups to native workers in the local labor market, expanding it from the labor market to cultural values and social norms, and extending this theory to foreign-born European populations.

### 1.1. Literature Review

Group conflict theory posits that competition for scarce resources may exacerbate intergroup hostility [32]. When competition is more pronounced, the feeling that one group’s well-being is in jeopardy because of strengthening another group and the threat posed by this group leads to more negative outgroup sentiments [33]. Ethnic competition theory transcended this idea for ethnic groups [34,35,36], uniting group conflict theory with social identity theory [37]. According to the latter, people divide themselves and others into social groups, getting their sense of worth from belonging to these groups. Group identity and ethnic intergroup competition over resources are two main factors influencing attitudes toward immigrants [13].

The ethnic competition model’s basic claim is that group identities and the competition between groups for benefits, resources, power, and incentives impact attitudes toward minorities and migrants [12,34,36]. A competitive environment intensifies negative outgroup assessments and positive in-group identification [33]. Competition for economic and social resources makes people feel threatened by other ethnic outgroups [30].

There is extensive literature on the threats posed by immigrants. Integrated Threat Theory (ITT) [38] distinguished between realistic and symbolic threats. Realistic threats include threats to the in-group’s physical well-being such as health, threats to their political and economic power, and threats to the in-group’s survival such as those posed by warfare [39]. Economic threat is a core element of realistic threat [40]. At the heart of economic threat is the sense of rivalry for limited resources, including jobs and housing, and the belief that these resources belong to the local population but are in danger from outsiders [12]. Symbolic threat stems from perceived disparities in morality, values, norms, and beliefs among groups [39]. Threat to culture is an important dimension of symbolic threat [29]; perception of cultural threat stems from the fear that foreign customs and values may be harmful to those of the national culture [12,41]. Some studies focused only on the core elements of realistic and symbolic threats –economic and cultural threats [14,42].

In societies with large differences between the population groups, people tend to identify themselves with certain groups [43]. In such a case, the competition for resources is perceived not as competition between individuals but between groups. Quillian [44] distinguished between two levels of threats: those that arise at the micro-level (from an outgroup individual to an in-group individual) and those that arise at the macro-level (from a group of newcomers to a group of local residents). Threats from the whole group generate prejudice, which, in turn, reinforces the competition for material or symbolic resources even more (value domination) [45].

Large groups or groups growing quickly, as well as groups that are perceived as distanced from the local population by culture, language, economic situation, political and cultural values preferences, or even physical or visible characteristics, are especially likely to be recognized as possible rivals [46]. Groups that are perceived as having a high chance of bringing changes to the status quo may pose higher threats [45,47]. The arrival of newcomers can be seen as a threat to “the comfort of cultural traditions” since the norms, beliefs, and symbols of the newcomers may conflict with those of the host society. This can result in dread of other cultures taking precedence over the way of life embraced by the in-group [41]. Issues of national identity stir up strong feelings in public opinion even more than issues of labor market competition [12].

Perceptions of one’s group’s income and economic situation strongly predict not only threats but also attitudes toward newcomers and willingness to accept them [2,28]. Gorodzeisky & Semyonov [13] found that people with low incomes were more likely to be exclusively opposed to new immigrants of the same race/ethnic group as to the country’s population, but income did not matter in predicting opposition to immigrants of different races/ethnicities. Immigrants are caught in a vicious cycle: when they do well economically, they are seen as potential competitors; when they do poorly, they are seen as a drain on the social security system [48].

Immigration opposition is a defensive response to outgroup dangers and challenges to the local population’s access to resources [13]. In the face of modern normative standards that reject biased attitudes, it may be psychologically inconsistent for people to support discriminative policy against newcomers based on prejudice. Therefore, to restore psychological coherence, there is a need to justify to oneself not welcoming newcomers [49]. This may be justified by the threats posed by newcomers. Many studies found that the threats prevail among more vulnerable low-income or discriminated population groups [15,44]; these characteristics are often linked to one another. Individuals in the lowest income categories with a bad economic situation reported both higher perceptions of economic and cultural threats than high-income individuals [8,14]. Because of feeling threatened, people with vulnerable economic and social positions usually have more negative attitudes toward newcomers [50]. Thus, threats may mediate relationships between income and feelings of being discriminated against to willingness to accept newcomers.

Both majorities and minorities may perceive realistic threats to their well-being [39] or symbolic threats to the in-group’s worldview [51]. The study by Van der Zwan, Bles and Lubbers [15] showed that ethnic competition also applied to people with immigrant experiences. Similar to natives, foreign-born people who were jobless, less educated, or held low-status employment felt more threatened by new arrivals [33,44,52]. The feeling among immigrants of belonging to a discriminated group is a mirror reflection of the prejudice of native-born people toward immigrants. When newcomers of the same race and ethnicity as veteran foreign-born groups come, these groups may suffer from so-called statistical discrimination. Statistical discrimination occurs when employers see workers as members of a certain ethnic group and pay them low wages based not on their individual productivity but on the average productivity of the ethnic group [43,53]. When there are many newcomers of certain ethnic groups, and they receive low wages, veterans who belong to this ethnic group may also be underpaid. Thus, foreign-born people may feel the threat from newcomers similar to the native-born population.

According to the economic approach, newcomers are not always perceived as threats. Borjas [31] divided the native population into competing and complementary groups to immigrants in the labor market. The income of the competing group is usually harmed due to immigration since they compete with newcomers for the same resources, but the complementary group benefits from the arrival of newcomers. For example, an influx of low-skilled immigrants may strengthen the competition with low-income and low-skilled native workers for non-qualified jobs, making them a “competing” group, but high-skill natives may gain from the arrival of low-income immigrants since they pay less for the services that newcomers provide, and those who hire newcomers can now specialize in jobs that better fit their skills, which makes them “complementary” [31]. Immigrants are often inclined to co-ethnic dealing and creating an ‘ethnic economy’ where immigrant entrepreneurs hire co-ethnic workers, producing ethnic products for co-ethnic clients [43]. In this case, immigrants’ businesses may benefit from arriving newcomers who are similar to them ethnically, speak the same language, and have understandable cultural codes. Thus, the theory of Borjas may be transcended to understanding how veteran immigrants who already live in a country feel about newcomers.

When part of a certain group in the population increases, it becomes more influential economically, culturally, and politically (i.e., immigrants may tend to vote for immigrant political parties [54]). This influence may decrease discrimination against this ethnic group and immigrants as a whole. On the other hand, the arrival of newcomers in high numbers and their increasing influence often threaten the native population, when veterans of similar ethnicity to newcomers may be associated by the local population with newcomers and, therefore, discrimination against veteran foreign-born groups may increase. The idea that immigrants might eventually take over the national culture and undermine the national values of the receiving society is the basis for the perception of cultural threat and rivalry with immigrants [55]. Veteran immigrants who are more integrated into the host societies usually feel a higher threat from newcomers, but this threat decreases when the proportion of newcomers with a similar culture is higher [15]. Thus, not only the native population may be competing or complementary to newcomers, as Borjas [31] defined, but also veteran immigrants may be competing or complementary to newcomers from either economic or cultural points of view.

Numerous studies found an association between prejudice and discrimination, threats, and willingness to accept immigrants [5,6,7,8,9,10,11,12]. Schlueter, Schmidt and Wagner [9] found that increased deliberate hostile behavior toward foreigners was caused by higher levels of perceived group threat, but not vice versa. Hostile behavior did not cause a higher perception of threat. In line with this finding, Burhan and van Leeuwen [42] revealed that individuals who perceived immigrants as less of an economic or cultural threat were more inclined to offer immigrants direct help and support for empowerment. Likewise, McLaren [8] found that in Europe, a threat to economic resources or cultural values posed by immigrants translated into a willingness to receive or expel them. Although both symbolic (such as differences in cultural values) and realistic (economic) threats were positively correlated with support for anti-immigration policies, the effect of an economic threat on opposition to immigration is higher than that of a symbolic one [11,13]. Threat perceptions are a kind of rationalization stemming from an anti-outgroup mindset, when discriminatory behavior against immigrants and willingness to expel are justified more, the more they are perceived as a threat [49].

The characteristics of newcomers also matter. Anti-immigration discourse frequently has overtly discriminatory connotations and draws a contrast between “good” immigrants—such as those from Canada, Ireland, or Poland—and stigmatized ethnic or racial groups—such as Hispanics [4,56]. Non-Western immigrant groups are sometimes represented as supporting cultural or religious customs that are (supposedly) at odds with the social mores of the Western European nations that welcome them [9]. Brader, Valentino and Suhay [2] found that when threats strengthen, stigmatized outgroups of immigrants experience higher opposition than unstigmatized groups. The fears of Europeans stem from more than just immigration from low-income, far-flung nations, and internal immigration from Europe should not be disregarded as a potential threat [14].

### 1.2. The Current Study

The literature review showed that the local European population, either native-born or immigrant, may be competing or complementary to newcomers, namely, may be harmed or, on the contrary, benefit from the arrival of new immigrants [31]. These harms or benefits may be either economic or cultural. Belonging to a competing or complementary group is shaped by income or belonging to the discriminated group. Therefore, these two factors (income and belonging to the discriminated group) may predict economic and cultural threats from immigrants, and these threats, in turn, may predict willingness to accept newcomers. Thus, economic and cultural threats mediate relationships between income and belonging to discriminated groups, and willingness to accept newcomers of various groups. The willingness to accept newcomers may differ for various groups of newcomers; thus, willingness to accept three groups of newcomers will be examined: (1) newcomers of the same race or ethnic group as most of the country’s population; (2) newcomers of a different race or ethnic group than most of the country’s population; (3) newcomers from the poorer countries outside Europe. 

Based on the literature review, the following hypotheses were formulated:

**H1.**  *The income of respondents will be negatively associated with the economic threat (H1.1) and the cultural threat from immigrants (H1.2).*

**H2.**  *The feeling of belonging to a discriminated group will be positively associated with the economic threat (H2.1) and the cultural threat from immigrants (H2.2).*

**H3.**  *The economic threat will be negatively associated with the willingness to accept newcomers of a different race or ethnicity (H3.1), of the same race or ethnicity (H3.2), or from poorer countries outside Europe (H3.3).*

**H4.**  *The cultural threat will be negatively associated with the willingness to accept newcomers of a different race or ethnicity (H4.1), of the same race or ethnicity (H4.2), from poorer countries outside Europe (H4.3).*

**H5.**  *Income will be positively associated with the willingness to accept newcomers of a different race or ethnicity (H5.1), of the same race or ethnicity (H5.2), and newcomers from poorer countries outside Europe (H5.3).*

**H6.**  *The feeling of belonging to a discriminated group will be negatively associated with the willingness to accept newcomers of a different race or ethnicity (H6.1), of the same race or ethnicity (H6.2), and from poorer countries outside Europe (H6.3).*

**Figure 1:**
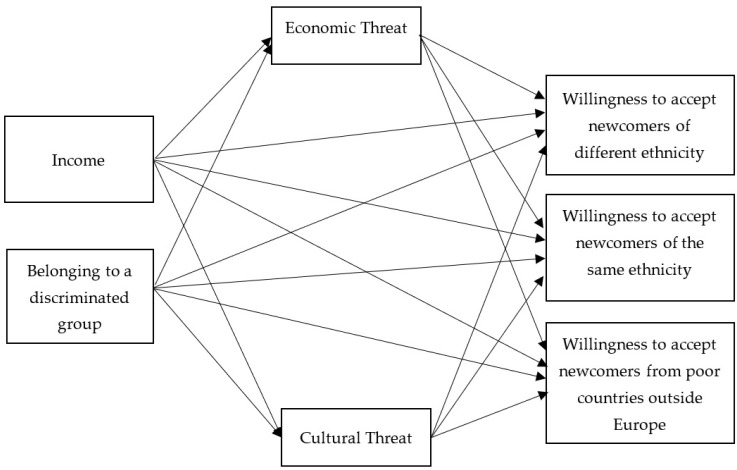
The study model.

The conceptual model of the study is presented in Figure 1. The model examines which factors shape the willingness of the local population to accept newcomers of various groups, and investigates whether being an immigrant themselves changes this willingness, differentiating between perceptions of three local groups: native-born population, EU immigrants, and non-EU immigrants.

## 2. Materials and Methods

The study used data from the European Social Survey Round 10 Data [57]. This is an academic cross-national survey conducted in European countries; the tenth round covered 31 countries. For this study, only respondents 18–65 years old living in the EU were chosen. The EU countries included in this round of the survey were Belgium (976 respondents), Bulgaria (1835 respondents), Czechia (1949), Estonia (1098), Finland (1046), France (1462), Greece (2157), Croatia (1143), Hungary (1324), Ireland (1215), Italy (1887), Lithuania (1227), Netherlands (1125), Portugal (1239), Slovenia (911), and Slovakia (975). In total, 21,569 respondents were chosen for the sample. Of them, 19,862 were native-born in the countries where they lived at the time of the survey, 1,136 were immigrants from non-EU countries (hereafter, non-EU immigrants), and 409 were immigrants from EU countries (born in one EU country and moved to another, hereafter EU immigrants). In all these countries, data collection was based on an hour-long face-to-face interview. Due to the pandemic, in some of these countries, some of the interviews were made by video. Round 10 fieldwork started in September 2020 and ended in August 2022.

Variables of willingness to accept newcomers were based on three separate questions: (1) “To what extent do you think [country] should allow people of a different race or ethnic group from most [country] people”, (2) “To what extent do you think [country] should allow people of the same race or ethnic group as most [country]’s people to come and live here”, and (3) “To what extent do you think [country] should allow people from the poorer countries outside Europe?”. All three variables were scaled from ‘1’ = allow none to ‘4’ = allow many to come and live here. Economic threat was based on the question: “Would you say it is generally bad or good for [country]’s economy that people come to live here from other countries?” and scaled from ‘1’ = bad for the economy to ‘11’ = good for the economy. Cultural threat was based on the question “Would you say that [country]’s cultural life is generally undermined or enriched by people coming to live here from other countries?” on a scale of ‘1’ = cultural life undermined to ‘11’ = Cultural life enriched. Total net household income from all sources was measured in deciles, from 1 to 10. Belonging to a discriminated group was a dichotomous variable where ‘1’ meant that a respondent described him/herself as being a member of a group that is discriminated against in his/her country on grounds of color, race, nationality, religion, language, or ethnicity. Sex was a dichotomous variable where ‘1’ meant being male. Age was measured in years, from 18 to 65. Education was scaled: ‘1’ = less than lower secondary; ‘2’ = lower secondary; ‘3’ = lower tier upper secondary; ‘4’ = upper tier upper secondary; ‘5’ = advanced vocational, sub-degree; ‘6’ = lower tertiary education, BA level; ‘7’ = higher tertiary education, MA level or higher. Employment was a dichotomous variable where ‘1’ meant being employed. Descriptive statistics of the variables are presented in Table 1.

## 3. Results

### 3.1. Descriptive Statistics

The income of households owned by native-born persons was slightly higher than that of EU immigrant households (*M* = 5.97, *SD* = 2.61 and *M* = 5.86, *SD* = 2.77, respectively), and the income of non-EU immigrant households was the lowest (*M* = 5.20, *SD* = 2.63). Non-EU immigrants reported more often that they were members of a group discriminated against on grounds of color/race/nationality/religion/language/ethnicity; 18.9 percent of them reported this vs. 6.5 percent of EU immigrants and only 2.7 percent of native-born respondents. Native-born people felt higher threat from immigrants to the economic situation and culture of their country (*M* = 5.95, *SD* = 2.55 and *M* = 5.74, *SD* = 2.64, respectively) than non-EU immigrants (*M* = 4.46, *SD* = 2.48 and *M* = 4.39, *SD* = 2.38 for economic and cultural threats, respectively) and EU immigrants (*M* = 4.36, *SD* = 2.50, *M* = 4.27, *SD* = 2.92, respectively).

The native-born population demonstrated the lowest willingness to accept newcomers to come and live in their country. The willingness of native-born people to accept newcomers of a different race or ethnic group from most of the country’s people was *M* = 2.49 (*SD* = 0.92) vs. willingness of non-EU immigrants *M* = 2.99 (*SD* = 0.82) and willingness of EU immigrants *M* = 2.92 (*SD* = 0.80). The same tendency was found for the willingness to accept newcomers from poor countries outside Europe: *M* = 2.43, *SD* = 0.93 for native-born people vs. *M* = 2.95, *SD* = 0.86 for non-EU immigrants and *M* = 2.84, *SD* = 0.86 for EU immigrants. The willingness to accept newcomers of the same race or ethnic group as most of the country’s people was slightly higher but, still, the native-born population showed the lowest willingness: *M* = 2.86, *SD* = 0.88 for native-born people, *M* = 3.15, *SD* = 0.75 for non-EU immigrants and *M* = 3.15, *SD* = 0.79 for EU immigrants.

### 3.2. Examination of Hypotheses

To examine the hypotheses, the multi-group Structured Equation Modeling (SEM) using AMOS 28 was run for three groups: native-born respondents, non-EU immigrants, and EU immigrants. SEM is appropriate for this study because it allows for the simultaneous analysis of several dependent variables within a single model. The fit indices of the model were acceptable and even good: *CFI* = 0.973, *χ^2^* = 32.377, *IFI* = 0.973, *NFI* = 0.973, *RMSEA* = 0.038, *SRMR* = 0.044. The model controlled for sex, age, education, and employment. The standardized effects of the model are presented in Table 2.

For native-born respondents, income was a determinant that significantly negatively predicted both economic and cultural threats (*β* = −0.121, *p* < 0.001 and *β* = −0.120, *p* < 0.001, respectively). For non-EU and EU immigrants, income effects were non-significant. Thus, Hypotheses 1.1 and 1.2 were supported only for the native-born group. Discrimination significantly negatively predicted economic threat (*β* = −0.072, *p* = 0.015) and cultural threat (*β* = −0.100, *p* < 0.001) for non-EU immigrants, but not for other groups. Since the found effects were negative, Hypotheses 2.1 and 2.2 that discrimination would be positively associated with economic and cultural threats were not supported.

Economic and cultural threats were negatively associated with the willingness to accept newcomers of different ethnicity from most of the country’s people, newcomers of the same ethnicity, and newcomers from poor countries outside Europe. Such negative effects were found for all three groups of respondents (see negative effects in Table 2). Hypotheses H4.1, H4.2, and H4.3 were supported for all three groups of respondents. 

Indirect effects of income on willingness to accept newcomers of different ethnicity from most of the country’s people (*β* = 0.075, *p* = 0.005), newcomers of the same race or ethnicity (*β* = 0.061, *p* = 0.007), and newcomers from poor countries outside Europe (*β* = 0.072, *p* = 0.004) were significant and positive for the native-born population. The indirect effects of belonging to a discriminated group on willingness to accept various groups of newcomers were non-significant. Namely, for this group, threats mediated only the relationships between income and willingness to accept newcomers. Since the direct effects of income on all three variables of willingness to accept newcomers were non-significant, threats fully mediated the relationships between them.

For non-EU immigrants, the indirect effects of belonging to a discriminated group on willingness to accept various groups of newcomers through economic and cultural threats were significant and positive (*β* = 0.046, *p* = 0.005 for accepting newcomers of different ethnicity from most of the country’s people; *β* = 0.037, *p* = 0.006 for accepting newcomers of the same ethnicity as most of the country’s people; *β* = 0.043, *p* = 0.008 for accepting newcomers from poor countries outside Europe). The indirect effects of income on willingness to accept various groups of newcomers were non-significant. Thus, for non-EU immigrants, threats mediated relationships only between belonging to a discriminated group and willingness to accept newcomers. Since the direct effects of belonging to a discriminated group on willingness to accept newcomers of different ethnicity from most of the country’s people and newcomers from poor countries outside Europe were non-significant, threats only partially mediated the relationships. For EU immigrants, all indirect effects were non-significant; no mediations were found.

The total direct and indirect effects (together) of income on willingness to accept newcomers of different ethnicity from most of the country’s people (*β* = 0.101, *p* = 0.009), newcomers of the same ethnicity (*β* = 0.092, *p* = 0.006), and newcomers from poor countries outside Europe (*β* = 0.090, *p* = 0.008) were significant and positive for the native-born population. For non-EU immigrants and EU immigrants, they were non-significant. Thus, hypotheses H5.1, H5.2, and H5.3 were supported only for the native-born population.

For non-EU immigrants, the total (direct and indirect) effects of discrimination on willingness to accept newcomers of different ethnicity from most of the country’s people were positive and significant (*β* = 0.123, *p* = 0.018), as well as the total effect of belonging to a discriminated group on willingness to accept newcomers from poor countries (*β* = 0.111, *p* = 0.011). For non-EU immigrants, direct effects between these variables were also significant and positive (*β* = 0.077, *p* = 0.025 and *β* = 0.068, *p* = 0.020, respectively). Thus, belonging to a discriminated group is a very important factor for predicting willingness to accept newcomers by non-EU immigrants. No significant total effect was found between belonging to a discriminated group and the willingness of non-EU immigrants to accept newcomers of the same ethnicity as the local population. For native-born persons and EU immigrants, no total significant effects between belonging to a discriminated group and willingness to accept any newcomers were found. Hypotheses H6.1 and H6.3 were supported for non-EU immigrants only. Hypothesis H6.2 was not supported for all population groups.

## 4. Discussion

This study showed how income and belonging to a discriminated group shaped perceptions of economic and cultural threats posed by immigrants and willingness to accept various groups of newcomers, and how immigrant experience and being ethnically distinct from the native population mattered in shaping these perceptions. The theoretical contribution of this study is three-fold. First, it justified that the integrated threat theory applies not only to the native population but also to the immigrant population in their attitudes toward other immigrant newcomers. For immigrant respondents in the EU, higher economic and cultural threats were associated with a lower willingness to accept newcomers. Second, it transcended Borjas’ [31] theory of competing or complementary to newcomer groups of native populations in the local economy, extending this theory to the cultural and social spheres, and showing how the local populations, either native-born or immigrant, may be competing or complementary to newcomers in non-economic spheres. Third, it showed which determinants, economic (income) or symbolic (belonging to a discriminated group), were more important to the native-born population and veteran immigrants in forming their position toward newcomers: income was more important for EU native-born people, and belonging to a discriminated group was more important for immigrants from non-EU countries. The study defined when a local group becomes competing or complementary to newcomers and what shapes this choice.

The study revealed that immigrants who arrived from EU and non-EU countries reported lower levels of threat posed by other immigrants to the national economy or culture than native-born people reported. They were also more likely than the native-born population to receive newcomers. This finding is surprising, since newcomers are usually perceived as competing for resources with local groups which are similar to them by ethnicity or immigrant experience. One possible explanation is that estimating the threat posed by newcomers to the European economy and culture as high may cause cognitive dissonance since the immigrant respondents were once newcomers; namely, they need to perceive themselves as threatening to host countries. The next surprising finding was that non-EU immigrants, who often differ by race and ethnicity from the native population, were more likely to receive newcomers of the same ethnicity as the native population than newcomers of different ethnicity from the native population. It seems that when immigrants integrate in a new country, they begin to identify themselves with the native population and apply their opinions and perceptions. This finding is in line with Van der Zwan, Bles and Lubbers [15], who found that the views of threats held by migrants, particularly those who had lived longer in the destination country and were better integrated, were positively correlated with natives’ opinions.

Although immigrants perceived lower levels of threat from other immigrants compared to the native population, those immigrants who did perceive higher threats reported a lower willingness to accept newcomers from various origins. Such negative effects of threats occurred also for the willingness of native people to accept newcomers of the same ethnicity as most of the country’s population, even to a slightly lesser extent compared to a willingness to accept newcomers of different ethnicity from most of the country’s population. The negative effects of threats on willingness to receive newcomers were stable for all respondents including native-born people, EU immigrants, and non-EU immigrants. Thus, threats worked similarly for native-born and immigrant groups. This is in line with Gorodzeisky and Semyonov [13], who defined threats as a universal and non-group-specific source of opposition to immigration that existed regardless of immigrant race, ethnicity, and origin. These findings justify that the integrated threat theory may be applied not only to natives but also to various veteran immigrant groups.

The study investigated two main determinants of threats and willingness to accept newcomers: income (economic determinant) and belonging to a discriminated group (symbolic determinant). The study revealed that these determinants varied across different population groups. For native-born respondents, the salient determinant was the income of their households; the native-born people whose income was higher reported lower levels of both economic and cultural threats. Income also predicted the willingness of the native population to accept newcomers of all groups, when higher income was associated with a higher willingness to accept. These relationships were mediated by economic and cultural threats posed by immigrants: native-born people of higher income reported fewer economic and cultural threats, and they were more likely to accept newcomers of either different ethnicity, the same ethnicity, or immigrants from poor countries. It may be explained by the fact that native-born people with higher incomes are less likely to compete with newcomers for economic resources and more likely to benefit from their arrival as business owners or consumers of newcomers’ cheap services. Transcending the theory of Borjas [31], native-born people with high income may be considered a group complementary to the newcomers from an economic point of view, and people with low income as a competing group. A feeling of being discriminated against did not matter for native-born people in predicting neither threats nor their willingness to accept newcomers.

For non-EU immigrants, the situation was the opposite. For them, belonging to a group that experienced racial, ethnic, national, or religious discrimination was much more important than income in predicting their perception of threats and willingness to accept newcomers. Those who felt they belonged to a discriminated group reported lower threats from newcomers and were more willing to accept them. Moreover, threats mediated these relationships: the feeling of being discriminated against was associated with lower levels of threats, and lower threats, in turn, were associated with a higher acceptance of newcomers of different ethnicity from most of the country’s people, the same ethnicity as most of the country’s people, or newcomers from poor countries outside Europe. One possible explanation may be developing social solidarity in response to discrimination. Experiencing discrimination can encourage people to look for solidarity and assistance from others experiencing similar challenges. In the face of discrimination, this solidarity might also embrace recent immigrants, creating a feeling of common identity and cause. One additional explanation is that non-EU immigrants who had felt discrimination benefited from the arrival of newcomers from a symbolic point of view. Increasing numbers of immigrants make this group more influential culturally and politically [54]; the rising impact and success of the group may reduce discrimination against its members. Thus, in terms of Borjas’s theory, non-EU immigrants who belong to a discriminated group may be complementary to newcomers. Non-EU immigrants who did not feel discriminated against were more threatened by newcomers and less likely to accept them. It seems that they identified themselves more with the native population, adapting its cultural and social norms, and therefore were a group competing with newcomers from the symbolic (cultural and social) point of view. For EU immigrants, whose income was higher than that of non-EU immigrants but lower than that of native-born people, and who were less discriminated against than non-EU immigrants, neither income nor discrimination played a role.

## 5. Conclusions

In sum, economic and cultural threats shape the willingness to accept newcomers similarly for native and veteran immigrant populations; this justifies that the integrated threat theory may apply also to immigrants and explains their perceptions toward other new immigrants. For the native-born group, the salient determinant of being competing or complementary to newcomers was an economic factor (income), and for non-EU immigrants, the salient factor was symbolic (belonging to a discriminated group). Thus, the theory of Borjas [31] may be applied not only in the economic and labor spheres but also may be expanded to symbolic cultural and social spheres. The determinants of being competing or complementary to newcomer groups vary not across the ethnicity of newcomers (different from or the same as most of the country’s population) but across the origin of the respondents themselves. Namely, being a non-EU immigrant, most of whom are of different race/ethnicity from most of the EU population and were discriminated against, matters more for predicting attitudes toward newcomers than ethnicity or economic situation in the newcomers’ home countries. Threats mediate these effects. Although in the literature competing groups are often represented by disadvantaged low-income and low-skilled people, and complementary groups by high-income and high-skilled well-established people, this study revealed that this is relevant only to the native-born population. Disadvantaged and discriminated groups of immigrants who are distinct from the native population may be complementary to newcomers, and the less discriminated and advantaged groups are competing. Thus, it is not necessarily a disadvantaged group that will compete with newcomers. Such a group may also be complementary to newcomers, depending rather on the origin of its members than on the origin of the newcomers.

## 6. Study Limitations

The data sample included respondents from sixteen EU countries. However, there was no distinction between respondents from different countries. This is because the purpose of the study was to understand how the EU population as a whole perceives newcomers. Understandably, differences across EU countries may occur, but this study grouped respondents by their origin (native-born, EU, and non-EU immigrant) and not by country. The second limitation is that the study did not address the length of immigrants’ living in the receiving country. The reason for this is that the study model was run among both native and immigrant population groups, and could not include variables relevant only to a part of the sample (only to immigrants). Future research in this area is therefore required. One additional limitation is that the European Social Survey’s Round 10 Data [57] fieldwork period spanned the years 2020 to 2022, during the COVID-19 pandemic. People in the EU may have responded in a biased manner as a result of its outbreak. Further research is needed under the new conditions of the influx of Ukrainian immigrants into EU countries after 2022.

## Figures and Tables

**Table 1:** Descriptive statistics.

Variables	Native-Born	Non-EU Immigrants	EU Immigrants
Income, scaled 1–10, Mean (*SD*)	5.97 (2.61)	5.20 (2.63)	5.86 (2.77)
Belonging to a discriminated group, %	2.7%	18.9%	6.5%
Economic threat, scaled 1–11, Mean (*SD*)	5.95 (2.55)	4.46 (2.48)	4.39 (2.38)
Cultural threat, scaled 1–11, Mean (*SD*)	5.74 (2.64)	4.36 (2.50)	4.27 (2.38)
Willingness to accept newcomers of different ethnicity from most of the country’s people, scaled 1–4, Mean (*SD*)	2.49 (0.92)	2.99 (0.82)	2.92 (0.80)
Willingness to accept newcomers of the same ethnicity, scaled 1–4, Mean (*SD*)	2.86 (0.88)	3.15 (0.75)	3.15 (0.79)
Willingness to accept newcomers from poor countries outside Europe, scaled 1–4, Mean (*SD*)	2.43 (0.93)	2.95 (0.86)	2.84 (0.86)
Male, %	46.6%		
Age, Mean (*SD*)	43.8 (13.4)	44.3 (12.9)	43.6 (12.1)
Education, %	100%	46.3%	46.0%
less than lower secondary	2.8	6.8	4.2
lower secondary	10.7	13.2	11.9
lower tier upper secondary	12.1	13.6	9.9
upper tier upper secondary	33.9	25.3	27.8
advanced vocational, sub-degree	10.4	10.1	10.7
lower tertiary education, BA level	15.1	11.8	13.2
higher tertiary education, MA level or higher	15.1	19.3	22.3
Employed, %	91.2%	91.9%	94.8%

**Table 2:** Standardized effects of the model (*β*).

Standardized Effects (*β*)	Groups of Respondents		
	Native-Born	Non-EU Immigrants	EU Immigrants
Direct effects:			
Income → Economic threat	−0.121 ***	0.008	−0.068
Income → Cultural threat	−0.120 ***	−0.028	−0.044
Belonging to a discriminated group → Economic threat	−0.001	−0.072 *	−0.031
Belonging to a discriminated group → Cultural threat	−0.009	−0.100 ***	−0.077
Economic threat → Willingness to accept newcomers of a different ethnicity from most of the country’s people	−0.290 ***	−0.293 ***	−0.301 ***
Economic threat → Willingness to accept newcomers of the same ethnicity as most of the country’s people	−0.263 ***	−0.230 ***	−0.229 ***
Economic threat → Willingness to accept newcomers from poor countries outside Europe	−0.291 ***	−0.340 ***	−0.243 ***
Cultural threat → Willingness to accept newcomers of different ethnicity from most of the country’s people	−0.335 ***	−0.253 ***	−0.257 ***
Cultural threat → Willingness to accept newcomers of the same ethnicity as most of the country’s people	−0.248 ***	−0.228 ***	−0.278 ***
Cultural threat → Willingness to accept newcomers from poor countries outside Europe	−0.327 ***	−0.186 ***	−0.262 ***
Income → Willingness to accept newcomers of different ethnicity from most of the country’s people	0.026	0.007	−0.040
Income → Willingness to accept newcomers of the same ethnicity as most of the country’s people	0.030	−0.032	0.034
Income → Willingness to accept newcomers from poor countries outside Europe	0.016	0.010	0.028
Belonging to a discriminated group → Willingness to accept newcomers of different ethnicity from most of the country’s people	0.012	0.077 **	0.064
Belonging to a discriminated group → Willingness to accept newcomers of the same ethnicity as most of the country’s people	0.006	0.020	0.041
Belonging to a discriminated group → Willingness to accept newcomers from poor countries outside Europe	0.008	0.068 **	0.038
Indirect effects:			
Indirect effect of Income on Willingness to accept newcomers of different ethnicity from most of the country’s people through Economic and Cultural threats	0.075 **	0.005	0.032
Indirect effect of Income on Willingness to accept newcomers of the same ethnicity as most of the country’s people through Economic and Cultural threats	0.061 **	0.004	0.028
Indirect effect of Income on Willingness to accept newcomers from poor countries outside Europe through Economic and Cultural threats	0.074 **	0.003	0.028
Indirect effect of Belonging to a discriminated group on Willingness to accept newcomers of different ethnicity from most of the country’s people through Economic and Cultural threats	0.003	0.046 **	0.029
Indirect effect of Belonging to a discriminated group on Willingness to accept newcomers of the same ethnicity as most of the country’s people through Economic and Cultural threats	0.003	0.037 **	0.029
Indirect effect of Belonging to a discriminated group on Willingness to accept newcomers from poor countries outside Europe through Economic and Cultural threats	0.003	0.043 **	0.028
Total effects:			
Income → Willingness to accept newcomers of different ethnicity from most of the country’s people	0.101 **	0.012	−0.009
Income → Willingness to accept newcomers of the same ethnicity as most of the country’s people	0.092 **	−0.028	0.062
Income → Willingness to accept newcomers from poor countries outside Europe	0.090 **	0.012	0.056
Belonging to a discriminated group → Willingness to accept newcomers of different ethnicity from most of the country’s people	0.015	0.123 **	0.094
Belonging to a discriminated group → Willingness to accept newcomers of the same ethnicity as most of the country’s people	0.008	0.057	0.070
Belonging to a discriminated group → Willingness to accept newcomers from poor countries outside Europe	0.011	0.111 **	0.066

* *p* < 0.05, ** *p* < 0.01, *** *p* < 0.001.

## Data Availability

Data of the ESS Round 10 Data [57] are available at https://doi.org/10.21338/NSD-ESS10-2020.
